# Supplementary material for: Assessment of Genetic Diversity for Drought, Heat and Combined Drought and Heat Stress Tolerance in Early Maturing Maize Landraces
Source: Plants (Basel). 2019 Nov 17;8(11):518. doi: 10.3390/plants8110518 (PMC6918211; doi:10.3390/plants8110518)
Supplement: Supplementary file 1 [file plants-08-00518-s001.zip › 616166supp/Supplementary Table S1.docx]

Supplementary Table S1: Genotypic and residual variance, and broad sense heritability estimates of grain yield (kg/ha) of the nine individual trials.

| **Statistic** | **Non-stress** | | |  | **Managed Drought stress** | |  | **Heat stress** | |  | **Combined drought and heat stress** | |
| --- | --- | --- | --- | --- | --- | --- | --- | --- | --- | --- | --- | --- |
|  | IK 2017 | IK 2018 | MK 2018 |  | IK 2017 | IK 2018 |  | KW 2018 | KW 2019 |  | KW 2018 | KW 2019 |
| Genotype | 908205.44 | 1336588.40 | 1057996.36 |  | 306984.41 | 402325.97 |  | 1010080.85 | 200335.87 |  | 172125.60 | 93040.26 |
| Residual | 325395.90 | 394315.84 | 3670155.02 |  | 314096.36 | 170645.21 |  | 780094.08 | 129434.07 |  | 263842.00 | 181711.91 |
| Replication | 2 | 2 | 2 |  | 2 | 2 |  | 2 | 2 | 2 | 2 |  |
| **Heritability** | **0.85** | **0.87** | **0.37** |  | **0.66** | **0.83** |  | **0.72** | **0.76** |  | **0.61** | **0.51** |

IK=Ikenne, MK=Mokwa and KW=Kadawa
