# Supplementary material for: Assessment of Genetic Diversity for Drought, Heat and Combined Drought and Heat Stress Tolerance in Early Maturing Maize Landraces
Source: Plants (Basel). 2019 Nov 17;8(11):518. doi: 10.3390/plants8110518 (PMC6918211; doi:10.3390/plants8110518)
Supplement: Supplementary file 1 [file plants-08-00518-s001.zip › 616166supp/Supplementary Table S2.docx]

**Supplementary Table S2:** Association of traits measured under optimal growing conditions with same traits under managed drought stress, heat stress and combined drought and heat stress, and those measured under managed drought stress with same traits under heat stress and combined drought and heat stress as well as with those measured under heat stress with the same traits under combined drought and heat stress.

| **Trait** | **OGC & MDS** | **OGC& HS** | **OGC & DSHS** | **MDS & HS** | **MDS & DSHS** | **HS & DSHS** |
| --- | --- | --- | --- | --- | --- | --- |
| Days to anthesis | 0.81^***^ | 0.68^***^ | 0.70^***^ | 0.75^***^ | 0.71^***^ | 0.84^***^ |
| Days to silking | 0.50^**^ | 0.76^***^ | 0.72^***^ | 0.60^***^ | 0.61^***^ | 0.84^***^ |
| Anthesis-silking interval | 0.32 | 0.41^*^ | 0.53^***^ | 0.07 | 0.23 | 0.54^***^ |
| Plant height | 0.67^***^ | 0.69^***^ | 0.67^***^ | 0.54^***^ | 0.50^**^ | 0.69^***^ |
| Ear height | 0.62^***^ | 0.44^**^ | 0.53^***^ | 0.68^***^ | 0.66^***^ | 0.67^***^ |
| Plant aspect | 0.56^***^ | 0.54^***^ | 0.49^**^ | 0.17 | 0.17 | 0.53^***^ |
| Husk cover | 0.16 | -0.11 | 0.16 | 0.04 | 0.01 | 0.40^*^ |
| Stay green | - | - | - | 0.08 | -0.03 | 0.11 |
| Leaf firing | - | - | - | - | - | 0.41^*^ |
| Tassel blast | - | - | - | - | - | 0.31 |
| Root lodging | - | - | - | -0.06 | -0.12 | -0.15 |
| Stalk lodging | - | - | - | 0.27 | 0.48^**^ | 0.06 |
| Ears per plant | 0.43^**^ | 0.37^*^ | 0.2 | 0.09 | -0.12 | 0.61^***^ |
| Ear aspect | 0.53^***^ | 0.27 | 0.08 | 0.12 | - | 0.59^***^ |
| Ear rot | 0.29 | 0.37^*^ | 0.27 | -0.002 | 0.27 | 0.32 |
| Grain yield | 0.52^**^ | 0.45^**^ | 0.23 | 0.21 | -0.01 | 0.69^***^ |

^*^ Significant at the 0.05 probability level.

^**^ Significant at the 0.01 probability level.

^***^ Significant at the 0.001 probability level.

OGC: Optimal growing conditions, MDS: Managed drought stress, HS: Heat stress, DSHS: Combined drought and heat stress
