# Supplementary material for: Assessment of Genetic Diversity for Drought, Heat and Combined Drought and Heat Stress Tolerance in Early Maturing Maize Landraces
Source: Plants (Basel). 2019 Nov 17;8(11):518. doi: 10.3390/plants8110518 (PMC6918211; doi:10.3390/plants8110518)
Supplement: Supplementary file 1 [file plants-08-00518-s001.zip › 616166supp/Supplementary Table S3.docx]

Supplementary Table S3: Mean grain yield and other traits of 36 maize accessions evaluated under optimal growing conditions, managed drought stress, heat stress and combined drought and heat stress between 2017 and 2019, in Nigeria.

| **Evaluation**  **Condition** | **AD**  **(days)** | **SD**  **(days)** | **ASI**  **(days)** | **PLHT**  **(cm)** | **EHT**  **(cm)** | **HC**  **(1-9)** | **SG**  **(1-9)** | **PASP**  **(days)** | **RL**  **(%)** | **SL**  **(%)** | **LF**  **(%)** | **TB**  **(%)** | **EPP** | **EASP**  **(1-9)** | **EROT**  **(%)** | **GY**  **(Kg/ha)** |
| --- | --- | --- | --- | --- | --- | --- | --- | --- | --- | --- | --- | --- | --- | --- | --- | --- |
| OGC | 55 | 57 | 2 | 189 | 100 | 4 |  | 4 | - | - | - | - | 0.81 | 5 | 0.23 | 3205.08 |
| MDS | 55 | 60 | 5 | 142 | 79 | 4 | 4 | 5 | 0.03 | 0.11 | - | - | 0.57 | 5 | 0.06 | 1749.17 |
| HS | 67 | 70 | 3 | 162 | 84 | 4 | 4 | 5 | 0.03 | 0.08 | 0.04 | 0.03 | 0.53 | 6 | 0.07 | 1443.00 |
| DSHS | 65 | 68 | 3 | 175 | 95 | 4 | 4 | 5 | 0.01 | 0.06 | 0.10 | 0.06 | 0.40 | 6 | 0.04 | 1088.39 |

AD= days to anthesis; SD= days to silking; ASI= anthesis silking interval; PLHT=Plant height, EHT=Ear height, HC= Husk cover; SG = Stay green characteristics; PASP=Plant aspect; RL= root lodging; SL = Stalk lodging; LF = Leaf firing; TB = Tassel blast; EPP= Ears per plant; EASP = Ear aspect; EROT = Ear rot; GY= Grain yield.

OGC: Optimal growing conditions, MDS: Managed drought stress, HS: Heat stress, DSHS: Combined drought and heat stress
