# Supplementary material for: Assessment of Genetic Diversity for Drought, Heat and Combined Drought and Heat Stress Tolerance in Early Maturing Maize Landraces
Source: Plants (Basel). 2019 Nov 17;8(11):518. doi: 10.3390/plants8110518 (PMC6918211; doi:10.3390/plants8110518)
Supplement: Supplementary file 1 [file plants-08-00518-s001.zip › 616166supp/Supplementary Table S4.docx]

**Supplementary Table 4:** Cluster means (base index values and other secondary traits) of 36 maize accessions evaluated under optimal growing conditions, managed drought stress, heat stress and combined drought and heat stress between 2017 and 2019, in Nigeria.

| **Trait** | **I** | **II** | **III** | **IV** | **V** |
| --- | --- | --- | --- | --- | --- |
| **Managed drought stress** | | | | | |
| Base Index | 14.98 | 5.43 | 1.41 | -4.33 | -12.82 |
| Grain yield (kg/ha) | 3901.61 | 2285.09 | 1881.75 | 1222.44 | 507.87 |
| Ears per plant | 0.94 | 0.71 | 0.60 | 0.49 | 0.23 |
| Ear aspect (scale:1-9) | 3 | 5 | 5 | 6 | 8 |
| Plant aspect(scale:1-9) | 3 | 5 | 5 | 6 | 7 |
| Stay green characteristics (scale:1-9) | 3 | 3 | 4 | 4 | 5 |
| Anthesis silking interval | 3 | 3 | 4 | 6 | 9 |
| Number of accessions | 1 | 11 | 11 | 9 | 4 |
|  |  | **Heat stress** |  |  |  |
| Base Index | 5.31 | -3.79 |  |  |  |
| Grain yield (kg/ha) | 2122.49 | 957.65 |  |  |  |
| Ears per plant | 0.69 | 0.41 |  |  |  |
| Ear aspect (scale:1-9) | 5 | 7 |  |  |  |
| Plant aspect(scale:1-9) | 5 | 5 |  |  |  |
| Stay green characteristics (scale:1-9) | 4 | 4 |  |  |  |
| Anthesis silking interval | 2 | 3 |  |  |  |
| Number of accessions | 15 | 21 |  |  |  |
|  | **Combined drought and heat stress** | | | | |
| Base index | 4.92 | -1.76 | -4.11 |  |  |
| Grain yield (kg/ha) | 1770.09 | 770.77 | 771.63 |  |  |
| Ears per plant | 0.60 | 0.31 | 0.29 |  |  |
| Ear aspect (scale:1-9) | 5 | 7 | 7 |  |  |
| Plant aspect(scale:1-9) | 5 | 5 | 6 |  |  |
| Stay green characteristics (scale:1-9) | 3 | 3 | 4 |  |  |
| Anthesis silking interval | 3 | 3 | 4 |  |  |
| Number of accessions | 12 | 14 | 10 |  |  |
